# Intersectin-1s deficiency in pulmonary pathogenesis

**DOI:** 10.1186/s12931-017-0652-4

**Published:** 2017-09-06

**Authors:** Niranjan Jeganathan, Dan Predescu, Sanda Predescu

**Affiliations:** 1 Rush University Medical Center, Chicago, IL 60612 USA; 2 Department of Pharmacology and Division of Pulmonary and Critical Care Medicine, Rush University, 1750 W. Harrison Street, 1415 Jelke, Chicago, IL 60612 USA; 3 Department of Pharmacology and Division of Pulmonary and Critical Care Medicine, Rush University Medical Center and Rush Medical College, 1750 W. Harrison Street, 1535 Jelke, Chicago, IL 60612 USA

**Keywords:** Intersectin-1s, Pulmonary arterial hypertension, Lung cancer, Acute lung injury, Eps8, mSos1, MAPK, Rac1

## Abstract

Intersectin-1s (ITSN-1s), a multidomain adaptor protein, plays a vital role in endocytosis, cytoskeleton rearrangement and cell signaling. Recent studies have demonstrated that deficiency of ITSN-1s is a crucial early event in pulmonary pathogenesis. In lung cancer, ITSN-1s deficiency impairs Eps8 ubiquitination and favors Eps8-mSos1 interaction which activates Rac1 leading to enhanced lung cancer cell proliferation, migration and metastasis. Restoring ITSN-1s deficiency in lung cancer cells facilitates cytoskeleton changes favoring mesenchymal to epithelial transformation and impairs lung cancer progression. ITSN-1s deficiency in acute lung injury leads to impaired endocytosis which leads to ubiquitination and degradation of growth factor receptors such as Alk5. This deficiency is counterbalanced by microparticles which, via paracrine effects, transfer Alk5/TGFβRII complex to non-apoptotic cells. In the presence of ITSN-1s deficiency, Alk5-restored cells signal via Erk1/2 MAPK pathway leading to restoration and repair of lung architecture. In inflammatory conditions such as pulmonary artery hypertension, ITSN-1s full length protein is cleaved by granzyme B into EH_ITSN_ and SH3A-E_ITSN_ fragments. The EH_ITSN_ fragment leads to pulmonary cell proliferation via activation of p38 MAPK and Elk-1/c-Fos signaling. In vivo, ITSN-1s deficient mice transduced with EH_ITSN_ plasmid develop pulmonary vascular obliteration and plexiform lesions consistent with pathological findings seen in severe pulmonary arterial hypertension. These novel findings have significantly contributed to understanding the mechanisms and pathogenesis involved in pulmonary pathology. As demonstrated in these studies, genetically modified ITSN-1s expression mouse models will be a valuable tool to further advance our understanding of pulmonary pathology and lead to novel targets for treating these conditions.

## Introduction

Intersectins (ITSNs) belong to the family of adaptor proteins [1]. Adaptor proteins, also known as scaffold proteins, mediate the interaction between receptors and signal transduction pathways by functioning as platforms for the assembly of multiple protein signaling complexes [2, 3]. Adaptor proteins lead to specificity in signaling via their sequence of protein domains/motifs, subcellular localization and their proximity to binding proteins [2, 3]. Thus, adaptor proteins play a crucial role in cell signaling in a spatial and temporal fashion, and regulate many important cellular processes including proliferation, differentiation, cell cycle control, cell survival and migration [2, 3]. Aberrant expression of adaptor proteins is implicated in numerous diseases [3].

ITSNs have a unique multi-domain structure each with a distinct ligand preference [4]. ITSNs were initially only associated with the regulation of endocytosis [5]. Subsequent studies have revealed a more complex role for these proteins in the regulation of cell signaling and cytoskeleton rearrangement [1, 6–10]. Recent studies implicate ITSNs, especially the transcript intersectin-1 short (ITSN-1s), in the pathogenesis of several pulmonary diseases [11–16]. Given these novel findings, this review article will provide a comprehensive overview of ITSN-1s’ regulation of biochemical pathways and its clinical implications in pulmonary pathology.

## Background

**Fig. 1:**
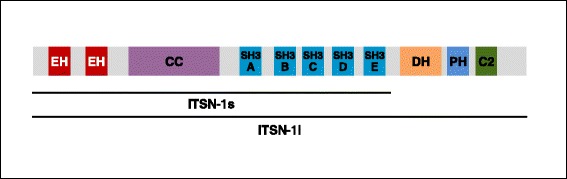
The domain structure intersectin-1 (ITSN-1) proteins. ITSN-1 proteins are comprised of Eps15 homology (EH) domains, a coiled-coil (CC) region, and 5 consecutive Src homology 3 (SH3) domains (SH3A, SH3B, SH3C, SH3D, SH3E), each with distinct ligands. A longer splice variant, termed ITSN-1 l, shares all the domains with the shorter spliced isoform, referred to as ITSN-1s, but in addition possesses a *C*-terminal extension encoding a Dbl-homology domain (DH), a pleckstrin homology (PH) domain and a C2 domain

The sequence and initial characterization of ITSN was first reported in Xenopus in 1998 by Yamabhai et al. [17]. They reported a 1270-amino acid long protein containing two Eps15 homology (EH) domains, a central coiled-coiled region, and five Src homology 3 (SH3) domains (Fig. 1). The protein was named ITSN because of its potential to bring together EH and SH3 domain binding proteins into a macromolecular complex [17]. ITSN was the first protein reported with both EH and SH3 domains which interacts with asparagine-proline-phenylalanine (NPF) and proline-rich (PXXP) motifs of the binding proteins, respectively [18].

Shortly thereafter, human ITSN was identified by Guipponi et al. [19]. ITSN exists with a high degree of similarity in a number of higher eukaryotes [20]. There are two ITSN genes in humans, ITSN-1 and ITSN-2, located on chromosome 21 and 2 respectively [19, 21]. ITSN-1 and ITSN-2 share identical domain structure and greater than 50% sequence identity [22, 23]. The human ITSN gene produces two main ITSN transcript mRNAs, short (ITSN-s) and long (ITSN-l), due to alternative splicing; human ITSN-s is 5.3-kilobases and ITSN-l is 15-kilobases [19]. Both ITSN-s and ITSN-l contain two EH and five SH3 domains [19, 21]. In addition, ITSN-l encodes for a Dbl homology (DH), a Pleckstrin (PH) and a C2 domain [19], Fig. 1. The EH domains bind to proteins associated with endocytosis [4, 17]. SH3 domains interact with proteins implicated in cell signaling and cytoskeleton organization [6, 7, 11, 13, 24, 25]. The DH domains promote guanine-nucleotide exchange on Rho, PH domains mediate interactions with inositol phospholipid and C2 domains mediate Ca2-dependent phospholipid binding [26].

ITSNs are widely distributed in human tissues, however the expression of ITSN isoforms is tissue-dependent; ITSN-1 l is specific to the brain and is absent in lung tissue whereas ITSN-1s, ITSN-2 s and ITSN-2 l are expressed ubiquitously [19, 21, 23]. Alternative splicing plays a major role in the regulation of ITSN gene expression, function and tissue specificity [4, 27]. In addition to the major splice variants there is a number of minor splice variants of ITSN-1s protein which facilitate tissue specific interactions [4, 19, 21]. This is illustrated in the fact that ITSN-1s preferentially interacts with mSos1 and Cbl in most tissues including lung [6, 11, 13, 28]. However, in brain tissue, a splicing of microexon 20 within the SH3A domain of ITSN-1s (resulting in inclusion of 5 additional amino acids) leads to reduced binding to mSos1 and Cbl, and enhanced interaction with CdGAP [29]. This transcript was conserved in numerous other eukaryotes examined. Additional splicing events have also been reported with ITSN-1s [4].

ITSN-1s is present in all subcellular compartments [8]. At the plasma membrane it is present in caveolae and clathrin-coated pits [8, 13, 26]. Activation of receptor tyrosine kinases (RTK) relocates ITSN-1s to the plasma membrane where it forms a complex with important cell signaling proteins [28]. Throughout the cytoplasm, ITSN-1s is associated vesicles, cytoskeleton elements and Golgi-like structures, in the perinuclear region [8, 13, 26]. Recent findings in our lab also show that ITSN-1s is present in the nucleus and interacts with important nuclear proteins (unpublished). The wide subcellular distribution of ITSN-1s is consistent with its involvement of multiple important signaling pathways involved in pulmonary pathogenesis.

### Role of ITSN-1s in Endocytosis and cell signaling

ITSN-1s plays an important role in endocytosis and vesicle trafficking; ITSN-1s binds to a number of important endocytic proteins and localizes to clathrin-coated pits and caveolae at the plasma membrane [5, 8, 30]. ITSN-1s binds several dynamin-2 molecules simultaneously and clusters them at endocytic sites creating a high concentration of dynamin-2, which is required for collar formation around the necks of endocytic vesicles, leading to membrane fission and endocytosis [5, 8, 31]. This interaction is via the SH3 domains of ITSN-1s [5, 8, 32]. The SH3A domain has the highest affinity for dynamin-2, and regulates its assembly-disassembly and the GTPase activity in the process of caveolae-dependent endocytosis [33]. However, ITSN-1s effects in regulating endocytosis are concentration-dependent as studies have shown that both silencing ITSN-1 gene (siRNA) and overexpressing ITSN-1s inhibit endocytosis [32, 33]. Overexpression of SH3A in endothelial cells (EC) as well as in mouse lung endothelium stimulates dynamin-2 assembly and stabilizes dynamin-2 oligomers preventing detachment of caveolae from the plasma membrane and as result, acting as a potent inhibitor of endocytosis [32, 33]. Similarly, acute depletion of ITSN-1s in mouse lungs results in inefficient dynamin-2 recruitment to the endocytic site leading to a decreased number of free caveolae. This impairs trans-endothelial transport which in turn opens interendothelial junctions and activates paracellular transport. Prolonged inhibition of ITSN-1s upregulates alternative endocytic structures/pathways which partially restore the junctional integrity [32].

Studies have also demonstrated that ITSN-1s regulates a number of important cell signaling pathways [1]. High-throughput yeast two-hybrid screening identified more than 100 interacting proteins with ITSNs (55 binding proteins for ITSN-1, 62 binding proteins for ITSN-2). In addition, ITSN-1 and ITSN-2 can dimerize with itself or each other [1]. Given the wide subcellular distribution of ITSN-1s, it has unique interactions and regulates different signaling pathways depending on the intracellular compartment and its spatial orientation.

Although ITSN-1s does not have a guanine nucleotide exchange factor (GEF) domain like ITSN-1 l, [C-terminal DH-PH domains act as a GEF [34, 35]], it plays an important role in the regulation of multiple Ras family GTPases [9, 10, 13]. Ras family proteins regulate many processes in the cell, including MAPK signaling, cell proliferation, cytoskeleton organization and cell migration [36]. Ras GTPases cycle between an active GTP-bound form and an inactive GDP-bound form [37, 38]. This cycling is regulated by GEFs that promote release of GDP and subsequent binding of GTP to activate Ras. GTPase activating or accelerating proteins (GAPs) enhance the intrinsic GTPase activity to promote GTP hydrolysis to terminate Ras GTPase activity [37, 39–42]. ITSN-1s interacts with CdGAP, mSos1 and Eps8 which regulate the activity of GTPase proteins Cdc42, Ras and Rac1 respectively [6, 9, 10, 13, 24, 43]. ITSN-1s’ interaction with CdGAP inhibits its GAP activity leading to activation of Cdc42 and Rac1 [24, 43]. This interaction is tissue/organ dependent; splicing of five additional amino acids in microexon 20 within the SH3A domain of ITSN-1s leads to enhanced interaction with CdGAP in brain tissue, and reduced binding in lung tissue [29].

ITSN-1s’ interaction with mSos1 is more complex. The SH3 domains of ITSN-1s form a stable complex with mSos1 and compete with Grb2 for binding to the same site on mSos1 [10, 12]. Activation of RTK recruits mSos1-Grb2 complex to activate Ras and MAPK pathway. Consistent with this, overexpression of the SH3 domains of ITSN alone impairs mSos1-Grb2 complex and prevents Ras-mediated MAPK activation [9, 10]. However, overexpression of full-length ITSN-1s activates Ras in the perinuclear vesicles without downstream activation of the MAPK pathway [6]. ITSN-1s mediated Ras activation in the perinuclear vesicles was initially thought to be via mSos1, but recent studies have disproved this observation and have implicated a novel PI3K isoform in this interaction [1]. The novel PI3K isoform, PI3KC2β, preferentially binds to inactive Ras and inhibits its activation. In the presence of ITSN-1s, the preferential interaction of PI3KC2β and ITSN-1s results in dissociation from inactive Ras which makes it available for immediate activation by GTP loading [44].

Eps8 interacts with mSos1 in the presence of RTK activation to convert the Rac1 GTPase from its inactive GDP-bound state to the active GTP-bound state [45]. ITSN-1s impairs mSos1-Eps8 interaction and favors Cbl-Eps8 interaction leading to impaired Rac1 activation and Eps8 ubiquitination, respectively. Cbl is an E3 ubiquitin ligase and plays an important role in regulating the levels of numerous proteins [46]. These interactions are most likely between the SH3 domain(s) of ITSN-1s and the proline-rich regions of Eps8 and Cbl, similar to ITSN’s interactions with most signaling proteins [22]. The multiple domains of ITSN-1s are known to simultaneously interact with the same protein as well as different proteins [5]. Given the presence of five SH3 domains, ITSN-1s is able to simultaneously interact with multiple proline-rich domains of proteins to coordinate different cellular signaling processes [7, 25]. Rac1 has a complex reciprocal relationship with RhoA, and the balance between the two determines cell migration by reorganizing cytoskeleton elements and focal adhesions [47, 48]. Consistent with this, impaired Rac1 activation in the presence of ITSN-1s leads to increased RhoA activation [13].

ITSN-1s also plays an important role in RTK’s regulation of numerous signaling pathways [28]. RTK function is regulated by endocytosis and ubiquitination. Although endocytosis terminates RTK signaling, it has emerged as a prerequisite step in the activation of signaling pathways [25, 49]. Internalization and trafficking of the epidermal growth factor receptor (EGFR) to endosomes is necessary for maximal activation of the MAPK pathway by EGFR [28]. Consistent with this, silencing ITSN-1s gene attenuates the extent and duration of Erk1/2 activation after epithelial growth factor stimulation, an effect that is due to decreased EGFR internalization [28]. ITSN-1s also decreases EGFR activity by promoting ubiquitination of EGFR by the ubiquitin E3 ligase Cbl [28]. In addition, ITSN-1s interacts with a number of other proteins involved in the regulation of Cbl such as sprouty2 and CIN85 [1, 50, 51].

### ITSN-1s deficiency in lung cancer

ITSNs’ participation in the activation of numerous mitogenic kinases provided strong suspicion for a potential involvement in cell proliferation and cancer. Earlier studies have shown that overexpression of ITSN-1s induces oncogenic transformation of rodent fibroblasts [52]. Both low and high levels of ITSN-1s have been reported in several cancers. The first clinical evidence for the role of ITSN-1s in cancer came from patients with Down syndrome [53]. The human ITSN1 maps proximal to the Down syndrome critical region (21q22.1-q22.2 between markers D21S319 and D21S65). Triplication (trisomy) of this region is associated with many phenotypes of Down syndrome [19]. As a consequence of ITSN-1s’ location in the down syndrome critical region, these patients have an elevated ITSN-1 mRNA and protein level [54]. In a cohort prospective registry study, these patients had a significantly lower incidence of solid organ cancers, especially lung cancer, compared to the age matched general population [53]. In addition, the Human Protein Atlas reports significantly lower levels of ITSN-1s protein level in lung cancer and a number of other solid organ malignancies [55]. However, studies in neuroblastoma and glioblastoma have shown upregulation of ITSN-1s and reduced tumorigenesis and cell migration with silencing of ITSN-1s gene [44, 56, 57]. This demonstrates that ITSN-1s mediated interactions and regulation of signaling pathways is specific to the tissue and disease. The numerous minor splice variants that occur throughout the ITSN-1s protein, results in changes in the EH, SH3, and DH domains and facilitate these tissue and disease-specific interactions [4]. Alternative splicing generates protein isoforms with different biological properties that differ in protein:protein interactions, subcellular localization, protein stability and posttranslational modifications [58]. ITSN isoforms have also been demonstrated to play a causative role in other diseases such as. glioblastoma, neuroblastoma, Alzheimer’s disease, Down syndrome, retinitis pigmentosa and tracheomalacia [44, 56, 57, 59–61]. It is increasingly recognized that the large number of alternative splicing events of ITSN-1s mRNA, the alternative polyadenylation, the tissue-specific expression level of different transcripts and their differential binding to interacting partners, play an important role in regulation of ITSN-1s in health and disease [27, 62, 63]. The detailed characterization of isoform specific-functions of ITSN-1s, and how specific changes in splicing impact the disease process and risk require further investigation.

The level of both ITSN-1s protein and mRNA is significantly decreased in human lung cancer likely due to suppression of transcription [13]. Pilot studies using immunohistochemistry staining in lung cancer tissue samples demonstrate a negative correlation between the ITSN-1s protein level and the aggressiveness of lung cancer. The subcellular distribution of ITSN-1s is not altered in lung cancer [13]. The impact of ITSN-1s deficiency in lung cancer progression was studied by comparing control lung cancer cells (A549) to those with ITSN-1s protein level restored to normal levels by stable transfection using a myc-ITSN-1s plasmid (A549 + ITSN-1s). ITSN-1s expression impairs lung cancer cell proliferation, anchorage-independent growth and tumorigenesis [13]. We have shown that ITSN-1s expression in lung cancer cells impairs Eps8 interaction with mSos1 and facilitates Eps8-Cbl interaction leading to ubiquitination and downregulation of Eps8. Eps8 interacts with mSos1 in the presence of RTK activation to convert the Rac1 GTPase from its inactive GDP-bound state to the active GTP-bound state [45]. Impaired Eps8-mSos1 interaction in the presence of ITSN-1s decreases Rac1 activation. Rac1 activation is a required for Ras-mediated tumor progression [64]. Rac1 activation also directly activates the JNK pathway leading to tumor progression [65]. As an oncoprotein, Eps8 also translocates to the nucleus and upregulates numerous cell cycle proteins such as transcription factor Foxm1 [66]. Therefore, ITSN-1s mediated inhibition of tumorigenesis is likely due to a combined inhibitory effect on these pathways. In addition, ITSN-1s enhances ubiquitination and downregulation of EGFR with a potential negative impact on proliferation [28].

In the presence of ITSN-1s, decreased Rac1 activation and reciprocal upregulation of RhoA shift the balance in favor of decreased lung cancer cell migration and metastasis [13]. This results in a number of significant changes in the cytoskeleton. In the presence of ITSN-1s, lung cancer cells show phenotypic changes favoring transition from mesenchymal-to-epithelial cells: increased spreading, lack of elongated and polarized morphology, and prominent actin bundles towards peripheral attachment points [13]. In addition, the number of vinculin focal adhesions at the cell surface is increased and the vimentin filament network is collapsed. In a scratch closure assay (in vitro cell migration model) ITSN-1 mediated cytoskeleton changes lead to significantly decreased cell migration and scratch closure. In vivo mouse metastasis assay shows significantly more tumor metastasis to the lungs as well as larger size tumors in A549 cancer cells compared to A549 + ITSN-1s cells. Altogether, these studies demonstrate that ITSN-1s deficiency is a crucial event in lung cancer progression. ITSN-1s’ ability to reverse the malignant features demonstrates the capability of this protein to regulate multiple pathways simultaneously which makes it an attractive therapeutic target. Further validation of ITSN-1s protein level in a large cohort of patients at different stages of lung cancer could establish ITSN-1s as a predictor of prognosis and indicator of response to therapy. Given the ubiquitous distribution of ITSN-1s and evidence that loss of ITSN-1s is a characteristic feature of many cancers [55], these findings may be applicable to other types of cancer.

### ITSN-1s deficiency in acute lung injury

Acute lung injury is associated with excessive apoptosis of pulmonary endothelial and epithelial cells, which induce endothelial and epithelial barrier dysfunction leading to pulmonary edema [67, 68]. Acute lung injury is triggered by direct injury to the lung and can be secondary to other inflammatory conditions such as sepsis, pancreatitis or transfusion rejection. Studies have shown that apoptotic pulmonary cells or macrophages engulfing apoptotic cells release substances such as growth factors, and create conditions that favor the emergence of apoptosis-resistant cells [68, 69].

In acute lung injury, as in many pro-inflammatory states, full-length ITSN-1s expression is decreased [11, 12, 68]. ITSN-1s is important for the pro-survival signaling pathway mSos1/Ras/Erk1/2 MAPK which regulates cell survival, proliferation and vascular remodeling [6, 9, 10]. Downregulation of ITSN-1s, via siRNA, inhibits the Erk1/2 MAPK pathway leading to mitochondrial apoptosis of EC [30]. However, EC with LPS-induced ITSN-1s deficiency do not undergo apoptosis since ITSN-1s deficiency is countered by upregulation of anti-apoptotic proteins leading to protection of EC [16].

The use of ITSN-1s knockdown (KD_ITSN_) mouse model has significantly advanced our understanding of the mechanisms involved in lung injury as well as the role of ITSN-1s in the pathogenesis of acute lung injury. KD_ITSN_ mouse model was generated by intravenous delivery of the cationic liposomes/siRNA_ITSN_ complex in repeated doses to maintain continuous downregulation of ITSN-1s protein expression up to 24 days. The amount injected (100 μg siRNA/mouse) was efficient and specific in inhibiting ITSN-1s mRNA and protein levels without significant adverse effects [11].

Acute KD_ITSN_ (assessed 72 h post-siRNA delivery) leads to decreased Erk1/2 MAPK signaling and causes significant EC apoptosis, microvascular loss and alveolar destruction. The lung injury is patchy and mostly involves the alveolar capillary units centered on small vessels and mid-sized vessels [11]. The morphology of ECs nuclei, mitochondria, and Golgi are affected as well. As a consequence, the alveolar-capillary unit becomes increasingly permeable leading to leakage of protein-rich fluid from the vascular to the interstitial space causing distension of the interstitial space [11]. These findings are identical to the pathophysiological processes that are hallmarks of acute lung injury [68]. This pattern of lung injury triggered by ITSN-1s deficiency persists up to about 10 days.

ITSN-1s interaction with endocytic proteins is crucial for normal endocytosis [8]. The pulmonary EC of KD_ITSN_ mice demonstrate impaired normal endocytosis which occurs via caveolin and clathrin-coated vesicles [12]. ITSN-1s deficiency leads to upregulation of alternative endocytic pathways (ECs display enlarged endocytic structures, membranous rings and tubules) to compensate for deficiency in normal endocytosis and vesicular [32]. Both endocytosis and vesicular trafficking are crucial for growth factor receptor signaling and activity of their downstream targets [70]. TGFβ is a ubiquitous and multifunctional cytokine which in the setting of lung injury expresses anti-inflammatory properties confined to the extent of septal injury and facilitates recovery [71]. Alk5 is a widely expressed transforming growth factor β receptor I (TGFβRI) which complexes with TGFβRII when activated by growth factor TGFβ, and activates Smad2 and Smad3 proteins via the canonical TGFβ signaling pathway [72, 73]. In normal EC, internalization of Alk5 via clathrin-coated vesicles leads to TGFβ-induced activation of Smad2/3 pathway which is then recycled to the plasma membrane. In contrast internalization of Alk5 by caveolae is directed to ubiquitin proteasome pathway [73, 74]. The alternative pathways upregulated by ITSN-1s deficiency express predominantly caveolin-1 and therefore alters the endocytic trafficking of Alk5 in favor of enhanced degradation [12]. Consistent with this, in acute lung injury Alk5 expression is significantly decreased up to 10 days; however after 10 days Alk5 expression starts to gradually recover and reaches normal levels by 24 days [11]. The recovery of Alk5 expression is via the effects of microparticles. Microparticles are 0.5-1.0 μm diameter in size and play a crucial role in the communication between different cell types in normal and pathological settings. They have double membrane morphology and store important bio-effectors which play a crucial role in the recovery of lung injury by inducing endothelial modifications via membrane fusion and paracrine effects [68, 75]. At day 10 of ITSN-1s deficiency when EC apoptosis and lung injury are at their peak, there is a significant increase in microparticles containing Alk5. In the setting of prolonged ITSN-1s deficiency these microparticles are able to interact and transfer Alk5/TGFβRII complexes to dysfunctional EC [11]. The expression of TGFβ is also significantly increased at 10 days when lung injury is at its peak. As a result of increased TGFβ and restoration of its receptor Alk5/TGFβRI, the Erk1/2 MAPK pathway is restored, and remaining non-apoptotic quiescent EC exhibit phenotypic changes toward hyperproliferation and apoptosis resistance leading to increased microvessel density, repair and remodeling of the lungs [11]. Within 2 weeks after severe injury, mouse lung function returned to normal state with little evidence of prior damage. A lung repair process characterized by EC proliferation and increased microvascular density was critical for the remarkable recovery. However, the typical TGFβ/ALK5 signaling is shifted from Smad2/3 activation towards a less common Ras/Erk1/2 MAPK pathway [12]. Since ITSN-1s associates with mSos1 in a complex that excludes Grb2, ITSN-1s deficiency increases mSos1 availability for Grb2 interaction and results in preferential formation of ALK5/mSos1/Grb2 signaling complex. This shifts the balance from Smad2/3-Erk1/2 towards Ras/MEK/Erk1/2 activation [12]. Despite continuous and efficient KD_ITSN_, apoptosis of EC starts to decrease after 10 days, and by 24 days has reached almost normal levels. A similar proliferative pattern was also seen with epithelial alveolar cells.

As the timeline of acute lung injury/acute respiratory distress syndrome (ARDS) related pathology is shared by both mice and humans, and since ITSN-1s deficiency is also a characteristic of human lung tissue of acute lung injury/ARDS patients [76], we applied a translational approach to study microparticles from the blood of ARDS patients. Similar to the mouse studies, we have identified a population of Alk5/TGFβRI-immunoreactive microparticles [76]. Flow cytometry and calibrated/counted beads used to quantify the microparticles indicated that the Alk5-positive microparticle population is more numerous in the ARDS patients compared to healthy controls, consistent with reports of elevated levels of microparticles in disease settings [77]. Flow cytometry and magnetic bead separation via biotin-conjugated Alk5 Ab and streptavidin magnetic beads demonstrated that these Alk5-positive microparticles are immunoreactive to CD73 and CD105 and negative to CD34 CD45, suggesting a mesenchymal stem cell origin [78]. Additional studies demonstrate that these particles interact with LPS-treated lung ECs, in culture and in vivo, leading to improved permeability and decrease in lung histological severity, consistent with long-term follow-up studies of ARDS survivors, suggesting that lung repair is in fact a hallmark of the normal course of recovery from acute lung injury [79]. Altogether, these observations demonstrate that extensive pulmonary EC death due to ITSN-1s deficiency stimulates mesenchymal stem cell paracrine signaling via microparticles leading to generation of hyperproliferative and apoptotic-resistant endothelial cells and subsequent recovery of lung injury.

### ITSN-1s deficiency in pulmonary arterial hypertension

Pulmonary arterial hypertension (PAH) is a disease in which there is persistent elevation of pulmonary artery pressure. The pathological findings in this disease are medial hypertrophy, intimal proliferation and fibrosis of pulmonary arteries and arterioles [80]. The intimal changes lead to progressive obstruction of the vessels which, in the presence of high pressure, dilate and evolve into microaneurysms. Areas of microaneurysms lead to endothelial proliferation and formation of in situ thrombosis which leads to the characteristic plexiform lesions. The obliteration of vessels and plexiform lesions lead to an increase in pulmonary vascular resistance and ultimately right heart failure. Enhanced proliferation of EC, smooth muscle cells and fibroblasts are central to the pathogenesis of PAH [81]. Emergence of proliferative ECs in PAH is a consequence of initial EC dysfunction and apoptosis and subsequent selection of apoptotic-resistant proliferative EC [82].

Inflammatory mechanisms play a significant role in the initiation of the pathogenesis of PAH. Inflammation associated with PAH attracts inflammatory cells to release granzyme B [83]. Interestingly, ITSN-1s is a substrate for granzyme B with a cleavage site at the IDQD^271GK^ sequence, a well-conserved sequence among mammals [84]. The cleavage results in decreased expression of full-length ITSN-1s protein and in two biologically active protein fragments, N-terminal fragment (EH_ITSN_) and C-terminal fragment (SH3A-E_ITSN_) [15]. Evidence demonstrates that ITSN-1s is a substrate for granzyme B and its cleavage leading to deficiency of ITSN-1s which is associated with the pathogenesis of PAH [15]. Mice treated with lipopolysaccharide (LPS; bacterial endotoxin which induces a strong immune response and leads to increase in granzyme B) and monocrotaline-induced PAH mouse and rat models results in loss of full-length ITSN-1s expression and the presence of a 28-kDa fragment corresponding to the molecular weight of EH_ITSN_ [15]. Biochemical analyses of lung ECs of PAH patients demonstrate low levels of full-length ITSN-1s protein and mRNA expression. Immunohistochemistry staining of human PAH specimens show lower ITSN-1s staining of pulmonary arteries with proliferative ECs and plexiform lesions whereas the presence of granzyme B is increased in the milieu of these lesions [15].

We have reported that both fragments formed by cleavage of full-length ITSN-1s are biologically active and impact EC proliferation [14, 15]. EH_ITSN_ enhances EC proliferation via activation of p38 MAPK pathway. In the presence of increased EH_ITSN_ expression, p38 MAPK is distributed predominantly in the cytosol and is activated. P38 MAPK activation leads to activation of Elk-1 transcription factor which facilitates Elk-1 binding to the c-Fos promoter leading to increased expression of the growth related protein c-Fos. Treatment with a selective p38 MAPK inhibitor significantly inhibits EC proliferation confirming the role of EH_ITSN_ mediated activation of p38 MAPK in EC proliferation. EH_ITSN_ has no effect on JNK or the PI3K/Akt signaling pathway, and impairs Erk1/2 MAPK activation via negative cross talk from p38 to Erk1/2. The SH3A-E_ITSN_ fragment, however, impairs proliferation by sequestering mSos1 and inhibiting activation of Ras/Erk1/2 MAPK signaling. The concurrent expression of both EH_ITSN_ and SH3A-E_ITSN_ results in a high p38/Erk1/2 MAPK activity ratio favoring EC proliferation [15].

ITSN-deficient mice transduced with EH_ITSN_ developed pathological findings similar to PAH patients. Wild-type mice and ITSN-deficient mice [KD_ITSN_ and ITSN knockout/heterozygous (K0_ITSN+/−_)] were treated with myc-EH_ITSN_ cationic lipoplexes delivered repeatedly by retro-orbital injection every 48 h for 20 days. No hypoxia or chemical/synthetic compounds known to induce PAH were used. EH_ITSN_ transduced ITSN-deficient mice developed numerous and widespread clusters of proliferating pulmonary ECs and pathological findings consistent with plexiform lesions; hypercellular and stalk-like lesions arising from the vessel walls protruded into the lumen of the pulmonary artery and caused severe obliteration of the vessel. These lesions displayed a rich matrix of collagen consistent with vascular medial thickening seen in human PAH [85]. Moreover, proliferation of pulmonary artery smooth muscle cells was also observed in the lung vessels of EH_ITSN_-transduced ITSN-1s deficient mice, suggesting significant cross-talk between ECs and smooth muscle cells leading to the pathogenesis of PAH which involves proliferation and hypertrophy of both intimal and medial layers. Only 20 days of EH_ITSN_ treatment, independent of any other insults, resulted in modest increase in the RVSP values (from 21 to 25.5 mmHg) and right ventricular hypertrophy.

Similar to the findings in cultured ECs, the lungs of EH_ITSN_-transduced ITSN-deficient mice showed increased activation of p38 MAPK, Elk-1 transcription factor and increased expression of the c-Fos gene, consistent with activation of p38 MAPK pathway [14]. Prior studies have also implicated p38 MAPK in cell proliferation and vascular obliteration leading to the pathogenesis of PAH [86]. Given that the motif NPF is an essential target of EH domains [87], the proliferative potential of ECs expressing EH_ITSN_ is impaired by treatment with a membrane permeable peptide containing the NPF motif [88]. As EH_ITSN_ is a highly specific molecular target, this could be a very effective treatment option to ameliorate and perhaps reverse the EC plexiform phenotype already established in severe human PAH.

## Conclusion

**Fig. 2:**
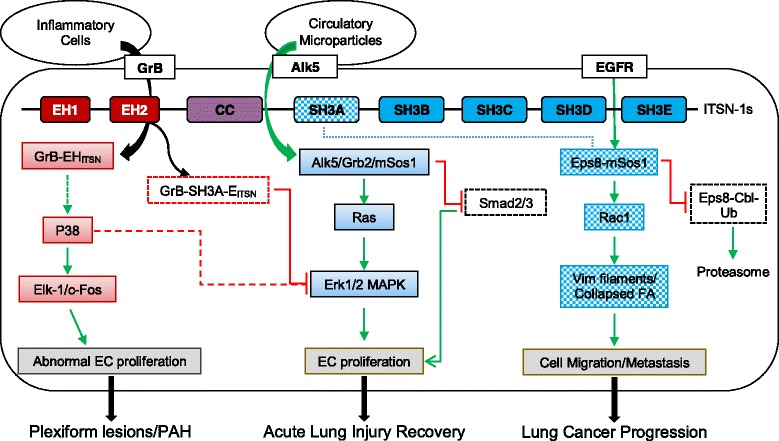
Schematic representation of molecular mechanisms involved in intersectin-1s (ITSN-1s) deficiency mediated pulmonary pathogenesis. The domain structure of full-length ITSN-1s is shown; EGFR, epithelial growth factor receptor; GrB, granzyme B; EC, endothelial cell; PAH, pulmonary artery hypertension; Ub, ubiquitin

ITSN-1s is a multi-domain protein with numerous binding partners capable of regulating many important signaling pathways. Studies to date implicate ITSN-1s deficiency in the pathogenesis of several pulmonary diseases (Fig. 2). In lung cancer, ITSN-1s deficiency shifts the balance in favor of greater Eps8-Sos1 interaction and less Eps8-Cbl interaction leading to activation of Rac1 and increased expression of Eps8 respectively. As a result of this and other potential interactions, ITSN-1s plays a crucial role in lung cancer development and progression: proliferation, anchorage-independent growth, cytoskeleton modification, migration and metastasis. As a pro-survival protein, ITSN-1s deficiency is a crucial early event in development of acute lung injury. ITSN-1s deficiency impairs normal endothelial structures and their function which leads to endocytosis of Alk5 receptors via alternative endocytic pathways resulting in Alk5 ubiquitination and degradation. Downregulation of Alk5 expression in acute lung injury is counterbalanced by circulating microparticles which, via paracrine effects, interact and transfer Alk5/TGFβRII complex to remaining non-apoptotic cells. These receptors, in a cellular context characterized by ITSN-1s deficiency and aberrant endocytosis, signal via Erk1/2 MAPK pathway, instead of the usual Smad 2/3 pathway, regardless leading to restoration and repair of lung architecture. In the setting of PAH, full-length ITSN-1s is cleaved by granzyme B released by inflammatory cells. The cleavage results in EH_ITSN_ and SH3A-E_ITSN_ fragments. The EH_ITSN_ fragment leads to EC proliferation via activation of p38 MAPK and Elk-1/c-Fos signaling. The SH3A-E_ITSN_ fragment impairs Ras/Erk1/2 MAPK signaling. However, the concurrent expression of both fragments results in high p38/Erk1/2 MAPK activity favoring pulmonary cell proliferation. In vivo, ITSN-1s deficient mouse transduced with EH_ITSN_ plasmid leads to EC and smooth muscle proliferation resulting in pulmonary vascular obliteration and plexiform lesions consistent with pathological findings seen in severe PAH. As shown in these studies related to pulmonary diseases and others, ITSN-1s regulates multiple signaling pathways simultaneously in a tissue, concentration and subcellular distribution specific manner. Given the numerous protein-protein interactions between the multiple domains of ITSN-1s, it is highly likely that additional regulatory pathways remain to be identified. Further studies will shed light into novel mechanisms of regulation of protein, genetics and epigenetics by ITSN-1s in pulmonary diseases. Future studies should also explore the role of other ITSN transcripts and potential compensatory role in pulmonary pathogenesis. Studies of ITSN-1s involvement in acute lung injury and PAH are limited and its role in the pathophysiology of these diseases is less established. The KD_ITSN_ and K0_ITSN+/−_ mouse models of lung injury as well as the EH_ITSN_-transduced K0_ITSN+/−_ mouse model of plexogenic PAH that recapitulate many of the pathological events associated with the human disease are valuable tools to further advance our understanding of ITSN-1s involvement in pulmonary pathology, and provide novel targets for treating these severe human conditions.

## Abbreviations

ARDS: Acute respiratory distress syndrome
DH: Dbl homology
EC: Endothelial cells
EGFR: Epidermal growth factor receptor
EH: Eps15 homology
EH_ITSN_ and SH3A-E_ITSN_: N-terminal fragment and C-terminal fragment
GAPs: GTPase activating or accelerating proteins
GEF: Guanine nucleotide exchange factor
ITSN-1s: Intersectin-1s
ITSNs: Intersectins
K0_ITSN+/−_: ITSN knockout/heterozygous
KD_ITSN_: ITSN-1s knockdown
NPF: Asparagine-proline-phenylalanine
PAH: Pulmonary arterial hypertension
PH: a Pleckstrin
SH3: Src homology 3
